# 

**Published:** 1914-08-15

**Authors:** 


					﻿THE
DENTAL
REGISTER
Edited by DrNELVILLE S. HOFF
ANN ARBOR, MICH.
VOL. LXVIII. AUGUST 1914 NO. 8
THE SAMUEL A. CROCKER CO., Publishers
OHIO DENTAL AND SURGICAL DEPOT
CONRAD BLDG., 18 & 20 W. Seventh St., CINCINNATI, O.
PRICE, ONE DOLLAR A YEAR
Entered at the Post-Offlce at Cincinnati, 0 , as second-class mall matter
				

